# Assessment of Collagen and Fibroblast Properties via Label-Free Higher Harmonic Generation Microscopy in Three-Dimensional Models of Osteogenesis Imperfecta and Ehlers-Danlos Syndrome

**DOI:** 10.3390/ijms262411848

**Published:** 2025-12-08

**Authors:** Yuanyuan Ma, Qiyu Bo, Zhiqing Zhang, Ludo van Haasterecht, Peter Kloen, Thomas Rustemeyer, Laura Ventura, Lidiia Zhytnik, Elisabeth M. W. Eekhoff, Dimitra Micha, Marie Louise Groot

**Affiliations:** 1 Department of Physics, LaserLab Amsterdam, Vrije Universiteit Amsterdam, 1081 HV Amsterdam, The Netherlands; yuanyuan.ma@vu.nl (Y.M.); ludovanhaasterecht@gmail.com (L.v.H.); 2 Institute of Modern Optics, Nankai University, Tianjin 300350, China; bob9876@mail.nankai.edu.cn (Q.B.); zhiqing.andy_zhang@nankai.edu.cn (Z.Z.); 3 Department of Orthopedic Surgery and Sports Medicine, Amsterdam University Medical Center, Location Meibergdreef 9, 1105 AZ Amsterdam, The Netherlands; p.kloen@amsterdamumc.nl; 4 Amsterdam Movement Sciences, 1081 BT Amsterdam, The Netherlands; l.ventura@amsterdamumc.nl (L.V.); l.zhytnik@amsterdamumc.nl (L.Z.); 5 The Netherlands Department of Dermato-Allergology and Occupational Health, Amsterdam University Medical Center, Location AMC, University of Amsterdam, 1000 GG Amsterdam, The Netherlands; t.rustemeyer@amsterdamumc.nl; 6 Amsterdam Reproduction & Development, 1105 AZ Amsterdam, The Netherlands; 7 Rare Bone Disease Center Amsterdam, 1081 HV Amsterdam, The Netherlands; emw.eekhoff@amsterdamumc.nl; 8 Department of Human Genetics, Amsterdam University Medical Center, Location Vrije Universiteit Amsterdam, 1081 HV Amsterdam, The Netherlands; 9 Department of Endocrinology & Metabolism, Amsterdam University Medical Center, Location Vrije Universiteit Amsterdam, 1081 HV Amsterdam, The Netherlands

**Keywords:** label-free, higher harmonic generation microscopy, three-dimensional, osteogenesis imperfecta, Ehlers–Danlos syndrome, collagen, fibroblast, connective tissue disorders, disease modeling

## Abstract

Osteogenesis imperfecta (OI) and Ehlers–Danlos syndrome (EDS) are inherited connective tissue disorders caused by diverse genetic defects, many of which affect collagen biosynthesis. However, the identified genetic variants do not always fully explain the clinical heterogeneity observed in patients, highlighting the need for advanced models and imaging techniques to assess collagen structure and fibroblast behavior at the microscopic level. In this study, we employed 5-week three-dimensional (3D) dermal fibroblast cultures derived from patients with haploinsufficient (HI) and dominant-negative (DN) OI, EDS, and healthy controls. Using label-free higher harmonic generation microscopy (HHGM), we visualized and quantified secreted collagen fibers and fibroblast morphology in situ. We analyzed fibroblast 3D orientation, collagen fiber diameter, collagen amount per cell, and the spatial alignment between fibroblasts and collagen fibers. HI OI fibroblasts secreted significantly less collagen than both control and EDS-derived cells, while EDS samples exhibited thinner collagen fibers compared to controls. Across all groups, collagen fiber orientation was strongly correlated with fibroblast alignment, in line with the role of fibroblasts in matrix organization. In healthy controls and HI OI samples, we observed a depth-dependent, counterclockwise rotation in fibroblast orientation from the culture bottom to the surface—a pattern that was less prominent in DN OI and EDS samples, potentially reflecting altered matrix guidance in diseased tissues. Overall, the quantity and quality of collagen, as well as fibroblast morphology and organization, were markedly altered in the OI and EDS model systems. These alterations may mirror tissue-level manifestations of the diseases, demonstrating the physiological relevance of patient-derived 3D fibroblast models for OI and EDS, as well as the power of harmonic generation microscopy in probing the cellular and extracellular consequences of disease-related gene defects in collagen or its biosynthetic pathways. Extensions of this methodological approach provide a way towards deeper understanding of tissue-level manifestations of collagen dysregulation in connective tissue disorders.

## 1. Introduction

Osteogenesis imperfecta (OI) is a heterogeneous group of skeletal dysplasia characterized by bone fragility and deformity, growth deficiency, and other secondary connective tissue defects [1,2]. OI is primarily caused by defects in the *COL1A1* and *COL1A2* genes, which encode the collagen type I protein. These pathogenic variants lead to either collagen deficiency, known as haploinsufficiency (HI), or structurally abnormal collagen, known as dominant-negative (DN) [3]. Ehlers–Danlos syndrome (EDS) is another inherited connective tissue disorder with overlapping features. It can arise from genetic pathogenic variants affecting collagen biosynthesis or structure, resulting in variable fragility in diverse soft tissues such as skin, arteries, bowels, and ligaments [4,5].

Collagen, particularly type I, is the principal structural protein in connective tissues, comprising approximately 60–90% of the dry weight of bone, skin, sclera, and tendons [6]. To understand how defects in collagen synthesis or structure contribute to OI and EDS pathology, experimental models must capture both molecular and structural aspects of collagen regulation in its native extracellular matrix (ECM) environment. Traditionally, collagen studies have relied on collagen electrophoresis from fibroblasts cultured in two-dimensional (2D) systems [7]. While informative about intracellular and extracellular fibrillar collagen chains, such simplified models do not replicate the complex 3D organization, cell–matrix interactions, or mechanical cues that govern collagen deposition in vivo. As a result, the relationship between fibroblast morphology, collagen secretion, and matrix architecture remains poorly understood.

To overcome these limitations, we previously developed a three-dimensional (3D) in vitro model using primary human dermal fibroblast cultures treated with ascorbic acid over 5 weeks to stimulate collagen secretion [3,8]. This model more closely mimics the spatial organization of native connective tissue, enabling us to study the effect of collagen gene pathogenic variants on both collagen secretion and matrix organization in OI and EDS. However, quantitative assessment of the relationship between fibroblast morphology and collagen architecture remains challenging due to the limited field of view and poor visualization of unlabeled cells in conventional confocal microscopy.

In the present study, we address these challenges by integrating our 3D fibroblast culture model with label-free higher harmonic generation microscopy (HHGM), a combination of second- and third-harmonic generation microscopy (SHG/THG). This approach enables high-resolution, three-dimensional, live visualization of both fibroblast morphology and secreted collagen within intact 3D matrices. HHGM provides deeper imaging penetration, a larger field of view, and maintenance of cell viability throughout imaging, while eliminating the need for staining or other perturbative processing steps [9].

By combing advanced 3D fibroblast culture models with label-free nonlinear optical imaging, this study introduces a powerful framework to investigate how disease-causing collagen mutations alter ECM architecture and cell–matrix organization in OI and EDS, illustrated through a first set of proof-of-concept experiments.

## 2. Results

### 2.1. HHGM Images: Qualitative Assessment of 3D Cell Cultures

#### 2.1.1. Visualization of Fibroblasts and Collagen in HHGM Images

**Figure 1:**
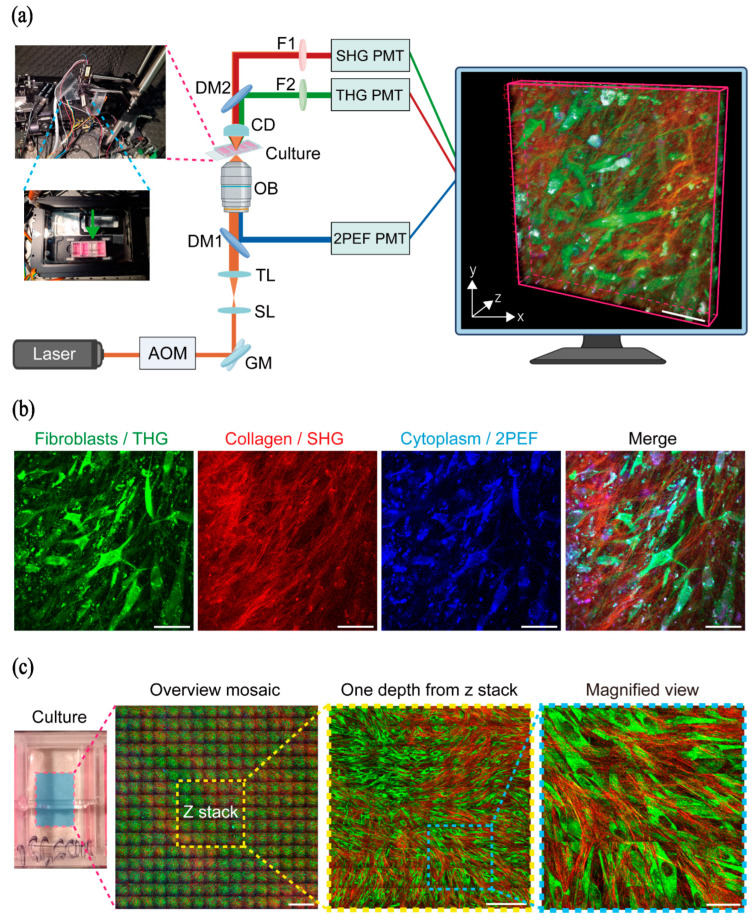
Experimental setup and label-free higher harmonic generation microscopy (HHGM) imaging of 3D fibroblast cultures. (**a**) Schematic of the label-free higher harmonic generation microscopy (HHGM) system. Components: AOM, acousto-optic modulator; GM, galvo mirror; SL, scan lens; TL, tube lens; DM, dichroic mirror; OB, 40×/1.30 NA oil immersion objective (Nikon, Tokyo, Japan); CD, aspheric condenser lenses; F1, second harmonic generation (SHG) filter; F2, third harmonic generation (THG) filter; PMT, photomultiplier tubes. Right: 3D rendering of a region of interest (ROI) from a z-stack acquired from a 5-week-old 3D control fibroblast culture (donor C2) using a portable epi-direction HHGM setup [10]. Imaging was performed in high-quality mode (4 pixels/μm) with a field of view (FOV) of 250 × 250 × 30 μm^3^ and 1 μm z-interval. Scale bar, 50 μm. Images partly created with BioRender.com. (**b**) Maximum intensity z-projection of a single cell layer from the z-stack shown in panel a, with individual channels for fibroblasts (THG), collagen (SHG), and cytoplasm displayed separately. Scale bar, 50 μm. (**c**) Overview image of a 5-week-old 3D fibroblast culture from control donor C2. A 4 × 4 mm^2^ mosaic was acquired in high-speed, lower-resolution mode (1.2 pixels/μm). A high-resolution z-stack (1 × 1 mm^2^ FOV, 2 μm z-step, 5 pixels/μm) was acquired within the mosaic area. Scale bars: 500 μm (mosaic), 200 μm (z-stack), and 50 μm (inset).

Fibroblasts, their secreted collagen, and cytoplasm can be clearly visualized using HHGM. The morphology of fibroblast cells and their nuclei is revealed by third harmonic generation (THG, shown in green), whereas secreted collagen is visualized by second harmonic generation (SHG, red), and the cytoplasm by two-photon-excited autofluorescence (2PEF, blue) (Figure 1a,b) [10]. For each well of the culture chamber, an overview mosaic covering a 4 × 4 mm^2^ area at the center was acquired. Subsequently, one or two regions of 1 × 1 mm^2^ were imaged in depth to obtain three-dimensional z-stacks (Figure 1c).

#### 2.1.2. Comparison of Fibroblast and Collagen Morphology

**Figure 2:**
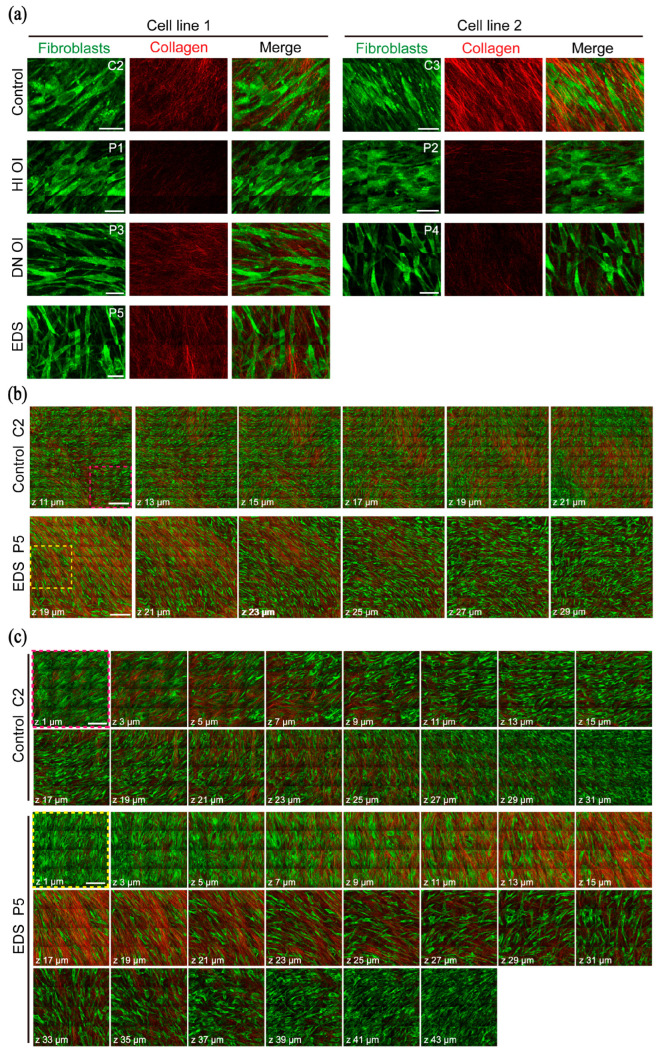
Fibroblast morphology and collagen organization across cell groups and representative z-stacks from control and EDS cultures. (**a**) Representative high-resolution HHGM images (5 pixels/μm) showing fibroblast morphology (THG) and their associated secreted collagen fibers (SHG) for each cell group. Two cell lines from each group (control, HI OI, and DN OI) are shown, except for EDS, which is represented by one line. Sample IDs are indicated in the top-right corner of each fibroblast image (e.g., C2: adult donor; C3: neonatal donor). Scale bars, 30 μm. (**b**) Selected depth-resolved HHGM images from z-stacks of a control (C2) and an EDS (P5) 3D fibroblast culture. Z-positions (distance from the culture dish bottom) are indicated for each image. All z-stacks used for quantification were acquired with a 1 × 1 mm^2^ FOV, 2 μm z-steps, and in high-quality mode (5 pixels/μm) using forward-detected THG and SHG signals. Scale bars, 200 μm. (**c**) Zoomed-in views of local regions from the z-stacks shown in (**b**), highlighting the main orientation of fibroblasts at each z-depth. Scale bars, 100 μm.

Comparison of the HHGM images of the healthy, HI OI, DN OI, and EDS groups revealed differences in fibroblast morphology and collagen deposition. Representative HHGM images illustrating fibroblast morphology and secreted collagen organization across the sample groups are shown in Figure 2a. Fibroblasts from the HI OI group exhibited a rounder shape compared to the more elongated morphology observed in control samples. In contrast, fibroblasts from the DN OI and EDS groups displayed narrower and more elongated shapes. The SHG images revealed that secreted fibers were primarily located adjacent to fibroblasts, with individual collagen fibrils clearly resolved. However, overall collagen deposition appeared reduced in fibroblasts derived from the disease groups (HI OI and DN OI) compared to controls.

#### 2.1.3. Counterclockwise Shift in Fibroblast Orientation

Analysis of local fibroblast organization revealed a consistent shift in the main cellular orientation. With increasing imaging depth across the z-stacks, both fibroblasts and collagen fibers gradually changed orientation, forming localized whorl-like patterns. Figure 2b displays depth-resolved HHGM images of fibroblast and collagen organization in 3D cultures from a control sample (adult donor C2) and an EDS sample (young donor P5). A magnified region (Figure 2c) highlights this counterclockwise rotational shift across successive z-layers, indicated by yellow arrows. Similar rotational patterns were observed in other cell lines (Supplementary Figure S1). Interestingly, this counterclockwise shift was less pronounced in regions with limited z-thickness (approximately 20–30 µm). In control samples, particularly in the neonatal donor sample (C3), this shift occurred more gradually compared to patient-derived fibroblasts (Supplementary Figure S1a). Additionally, z-stacks from control samples displayed denser networks of fibroblasts and collagen fibers, whereas the DN OI and EDS groups showed reduced cellular and collagen density. In contrast, the HI OI group exhibited densely packed fibroblasts but a noticeably sparser collagen matrix.

### 2.2. Quantification of HHGM Images

#### 2.2.1. Cell Density

**Figure 3:**
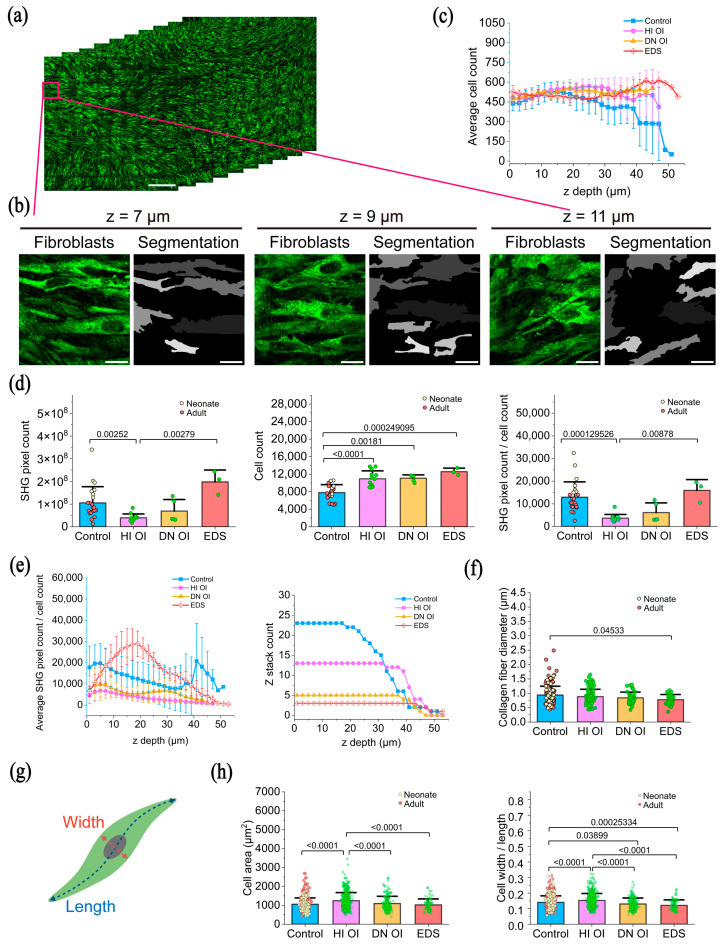
Analysis of collagen properties and fibroblast morphology from HHGM images. (**a**) Representative portion of a THG z-stack showing fibroblasts across a partial z-range. Scale bar, 200 μm. (**b**) Segmentation of all fibroblasts in all THG z-stacks. A zoomed-in region shows three contiguous cell layers at increasing z-depths from the culture dish bottom. In the segmentation image, each color corresponds to one segmented fibroblast. Scale bars, 20 μm. (**c**) Average fibroblast cell count per z-layer across all z-stacks in each cell group. (**d**) Quantification of total collagen amount (SHG signal), total fibroblast cell count, and collagen amount secreted per fibroblast across the four cell groups. Neonatal and adult controls are distinguished by light yellow and pink, respectively. Each point represents data from one z-stack. Data are shown as mean ± SD. SHG pixel count: Kruskal–Wallis ANOVA followed by Dunn’s test; Cell count: One-way ANOVA followed by Tukey’s test; SHG per cell: Kruskal–Wallis ANOVA followed by Dunn’s test. (**e**) Depth-resolved average collagen amount per fibroblast in each cell group, with the number of z-stacks contributing to the average at each depth also indicated. (**f**) Collagen fiber diameter across the four cell groups. Each point represents one individual fiber measurement. Neonatal and adult controls are shown in light yellow and pink, respectively. (Kruskal–Wallis ANOVA followed by Dunn’s test). (**g**) Schematic showing how fibroblast morphology was quantified, including manually traced cell area (green), central nucleus (gray), and cell width and length. (**h**) Quantification of fibroblast area and width-to-length ratio across the four groups. Neonatal and adult controls are shown in light yellow and pink, respectively. (Kruskal–Wallis ANOVA followed by Dunn’s test).

Cell density as a function of depth across the four groups was quantified using an automated cell segmentation algorithm applied to the THG image stacks. Figure 3a,b show representative segmentation results, with the original THG images on the left and the segmented fibroblasts (highlighted in gray) on the right. The average cell density was approximately 500 cells/mm^2^; however, it may be noted that this likely represents an overestimate, as the segmentation algorithm frequently identified partial cells.

Cell counts per z-stack for all groups—control, HI OI, DN OI, and EDS—are shown in Figure 3c as a function of depth from the bottom of the culture dish. In the first 40 µm of depth, all groups showed counts between 375 and 600 cells per z-stack. Fibroblasts located near the bottom surface of the chamber (in contact with the stiff glass substrate) appeared larger, consistent with previous reports [11], and were therefore excluded from further morphological analysis.

#### 2.2.2. Collagen Content

Statistical comparisons of total collagen amount and collagen per fibroblast count as a function of depth revealed marked group differences (Figure 3d). The total collagen content per z-stack was significantly reduced in the HI OI and DN OI groups—(4.0 ± 1.7) × 10^7^ pixels per z-stack and (7 ± 5) × 10^7^ pixels per z-stack, respectively—compared to the control and EDS groups, which showed (10 ± 7) × 10^7^ and (20 ± 5) × 10^7^ pixels per z-stack, respectively. Here, “pixels per z-stack” is defined as the number of SHG pixels above the defined intensity threshold within a 1 × 1 mm^2^ FOV and a z-depth of ~20 to 40 µm.

The total fibroblast count per z-stack was lowest in the control group (7800 ± 1800 cells), relative to the HI OI (11,000 ± 1800), DN OI (11,080 ± 750), and EDS (12,600 ± 800) groups. Nevertheless, the control fibroblasts secreted significantly more collagen per cell (12,900 ± 6800 pixels per cell) than those in the HI OI (3700 ± 1700) and DN OI (6100 ± 4300) groups. Interestingly, neonatal control fibroblasts secreted more collagen per cell than adult controls. Here, “cells per z-stack” denotes the fibroblast cell count within a volume of 1 × 1 mm^2^ FOV and z-depth ~20 to 40 µm.

Depth-resolved collagen secretion per fibroblast is shown in Figure 3e. Notably, in contrast to the other groups with fairly constant collagen production, in the EDS group, collagen was concentrated at intermediate depths.

#### 2.2.3. Diameter of Collagen Fibers

Collagen fiber diameter varied significantly between groups. In the EDS group, fibers were significantly thinner (0.78 ± 0.18 µm) than in the control group (0.94 ± 0.31 µm), whereas no significant differences were observed in the HI OI (0.89 ± 0.25 µm) and DN OI (0.84 ± 0.20 µm) groups compared to the control fibroblasts. Adult control cultures formed thicker fibers than neonatal controls (Figure 3f).

#### 2.2.4. Fibroblast Morphology

Fibroblast morphology was quantified by measuring cell area and width-to-length ratio based on the segmented outlines (Figure 3g). Fibroblasts from the HI OI group exhibited a larger average area (1250 ± 430 µm^2^) and a rounder shape (width-to-length ratio 0.15 ± 0.04) than fibroblasts from the control (1060 ± 330 µm^2^; 0.14 ± 0.04), DN OI (1090 ± 380 µm^2^; 0.13 ± 0.04), and EDS (1020 ± 310 µm^2^; 0.12 ± 0.04) groups (Figure 3h). Conversely, fibroblasts from the DN OI and EDS groups exhibited significantly narrower shapes compared with controls.

A negative correlation (r = −0.8, Matlab corr2 function) was observed between average cell area and average SHG pixel count across the four groups, indicating that the larger fibroblasts secreted less collagen (Supplementary Figure S2a).

#### 2.2.5. Three-Dimensional Orientation of Fibroblasts and Collagen

**Figure 4:**
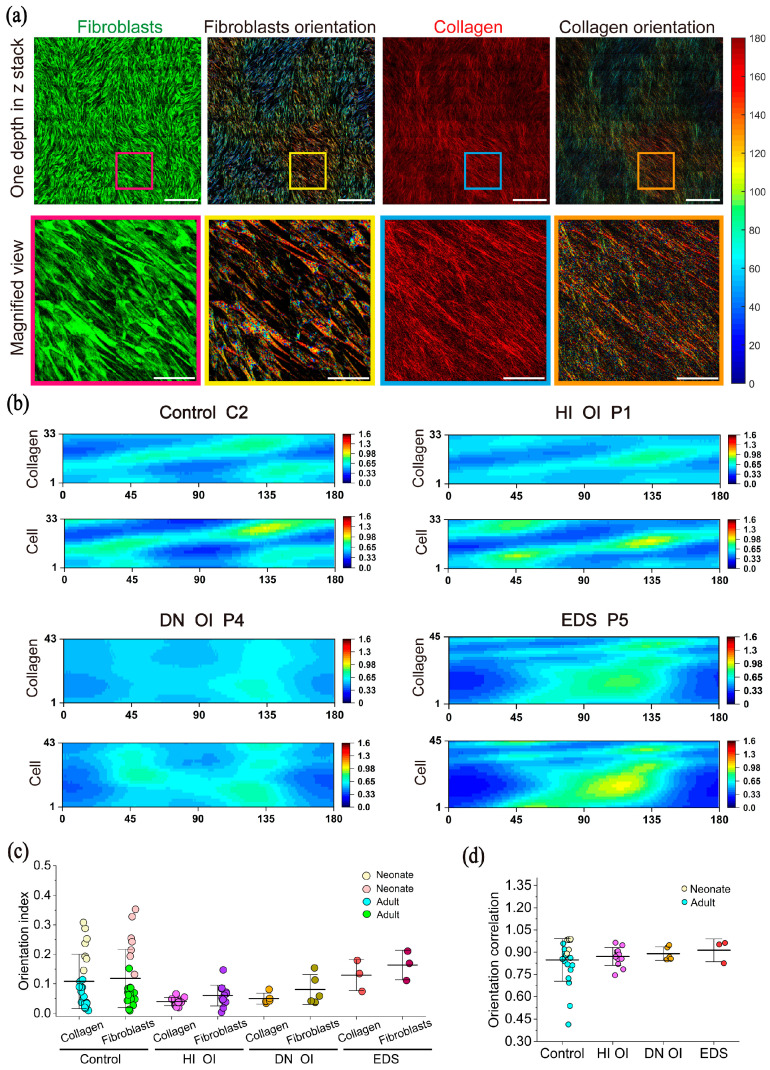
Three-dimensional orientation analysis of fibroblasts and collagen. (**a**) Representative HHGM images showing fibroblasts (THG) and collagen (SHG) (left), alongside the corresponding orientation colormap (right), where each pixel is color coded based on its ϑ angle (0–180°) according to the color bar. Scale bars: 200 μm (full FOV) and 50 μm (inset). (**b**) Representative ϑ orientation heatmaps of fibroblasts and collagen for the four cell groups: control (donor C2), HI OI (donor P1), DN OI (donor P4), and EDS (donor P5). Orientation is shown as the percentage of pixel count per angle (*x*-axis: ϑ angle 0–180°) across z-layers (*y*-axis: depth from culture dish bottom at 2 μm intervals). Red indicates higher pixel counts at a given angle, as shown on the color scale. (**c**) Three-dimensional orientation index of fibroblasts and collagen, calculated from both ϑ and φ angles for each pixel across the z-stacks. A value near 0 indicates disordered orientation, while a value near 1 indicates high alignment. Neonatal and adult controls are distinguished by light yellow/pink and blue/green, respectively. Each data point represents a single z-stack. Data shown as mean ± SD. (**d**) Correlation analysis between the ϑ orientation heatmaps of fibroblasts and collagen for each z-stack. Each point represents the correlation coefficient of a single z-stack; higher values indicate stronger alignment between fibroblast and collagen orientation. Neonatal and adult controls are shown in light yellow and blue, respectively. Data shown as mean ± SD.

The 3D orientations of fibroblasts and collagen were quantified and compared to assess alignment. Pixel-wise orientation angles (θ) were computed for both THG (fibroblasts) and SHG (collagen) images, assigning each pixel a unique color corresponding to its angle (0–180°) [12]. Representative THG and SHG images and their orientation maps are shown in Figure 4a. In the magnified region, both fibroblasts and collagen fibers exhibit a predominant orientation of ~140°, demonstrating a high degree of orientation correlation between the two components.

We further visualized depth-dependent orientation patterns as heatmaps for all cell groups (Figure 4b, Supplementary Figures S3 and S4). For each z-layer, pixel counts at each angle were normalized and expressed as percentage distributions. In both the control and HI OI groups, we observed a continuous shift in orientation from the culture bottom to the top layers. This shift was more gradual in neonatal controls (C3 and C4) than in adult controls (C1 and C2). In contrast, the DN OI and EDS groups exhibited less consistent orientation shifts, with more random and broader θ distributions across z-layers.

To quantify the overall alignment of collagen fibers and fibroblasts, we calculated a 3D orientation index based on both θ and φ angles for each pixel. An index close to 1 indicates high alignment, whereas values near 0 reflect disordered or depth-varying orientation. As shown in Figure 4c, the control group (collagen orientation index 0.11 ± 0.09; fibroblasts orientation index 0.12 ± 0.10) displayed a broad distribution of orientation indices, largely due to greater alignment in the neonatal lines (C3, C4) compared to adult controls (C1 and C2). The HI OI group (collagen: 0.04 ± 0.01; fibroblasts: 0.06 ± 0.04) and also the DN OI group (collagen: 0.05 ± 0.02; fibroblasts: 0.08 ± 0.05) exhibited the most disordered organization, whereas EDS (collagen: 0.13 ± 0.05; fibroblasts: 0.16 ± 0.05) group showed higher alignment patterns.

Finally, to assess the coupling between fibroblast and collagen orientation, we computed the correlation coefficient between their 2D orientation heatmaps (Figure 4d). All groups demonstrated strong correlation (mean R > 0.84), indicating a tight association between fibroblast and collagen orientation. However, the control group included a few samples with lower correlation values of 0.41 and 0.54. Further stratification showed that neonatal control cell lines had higher orientation correlation between fibroblasts and collagen than adult controls.

## 3. Discussion

In this study, we employed 3D primary dermal fibroblast cultures from OI and EDS patients, combined with label-free HHGM, to investigate how disease-causing collagen-related gene pathogenic variants affect fibroblast morphology, collagen deposition, and matrix organization in vitro. This advanced approach overcomes the limitations of conventional 2D cultures and confocal microscopy by enabling high-resolution, volumetric imaging of live fibroblast–collagen interactions in a physiologically relevant 3D context.

OI and EDS are genetic connective tissue disorders caused by collagen or collagen-related gene alterations [1,2,3,4,5]. Patient-derived dermal fibroblasts serve as valuable in vitro models for studying connective tissue disorders, as skin biopsies are minimally invasive and fibroblasts have a primary role in collagen and ECM synthesis [13]. However, these cell models lack measurements in 3D culture conformation which our study aims to address.

Our findings reveal distinct and disease-consistent alterations in both fibroblast morphology and collagen organization. In particular, fibroblasts from the HI OI group appeared rounder and secreted significantly less collagen, consistent with the primary deficiency in collagen type I production due to reduced *COL1A1* gene dosage. Interestingly, fibroblast shapes from the control, DN OI, and EDS groups were more elongated; considering that these cell lines produced more collagen than HI OI, this may attribute the larger area of HI OI cells to their lesser collagen-producing output. However, the lack of clear correlation between the total collagen amount and cell area across the different genetic backgrounds can point to additional factors regulating cell morphology, such as the retention of certain migratory or contractile features [14]. Substrate stiffness is also known to determine cell area with cells showing a spreading morphology on a stiff substrate and a rounder appearance on softer supporting environments [15,16]. In our research, the borosilicate glass dish bottom provided a stiff substrate, with cell layers near the bottom showing a similar trend. A previous study did not identify significant differences in the effective Young modulus between the different cell groups in this model [3]. However, it must be noted that a higher ascorbic acid concentration was employed in our study which may have affected the resulting ECM lattice stiffness.

Beyond total collagen output, we observed differences in collagen fiber diameter, with the EDS group displaying significantly thinner fibers than controls. Thinner collagen fibers may contribute to the mechanical fragility of connective tissues in EDS, as reduced fiber thickness compromises tensile strength and matrix cohesion. This aligns with previous findings showing that collagen type I defects in OI can result in thinner collagen fibrils, decreased mechanical anisotropy of cortical bone, and an overall weaker, more brittle bone structure [17]. Similarly, studies have shown that collagen fibril diameter can vary significantly across EDS subtypes [18]. For instance, the recently described vascular *TBHS2* EDS variant associated with prolonged bleeding, age-related aortic dilation, and rupture showed a highly irregular and dense ECM in the reticular dermis with thin and disorganized collagen fibers [19]. Our findings about decreased collagen fiber diameter in EDS patients with *PLOD1* defects align with the mild fibril narrowing reported in aortic tissue in PLOD1-caused familial thoracic aortic aneurysms and dissections, a common feature with kyphoscoliotic EDS [20]; similarly to our results, collagen content was unaffected [21]. In addition to *PLOD1*, kyphoscoliotic EDS is also known to be caused by pathogenic variants in *FKBP14*. Despite its proposed role in collagen regulation, collagen from FKBP14 probands showed normal electrophoretic mobility and collagen fiber diameter was normal in skin tissue [22]. Although abnormal collagen diameter is considered a characteristic of OI and EDS pathology, conclusions cannot be extrapolated about disease severity [23,24]; this can be attributed to the scarcity and small scale of such studies. Moreover, variations in collagen composition are known in different tissues, highlighting the need for standardized models and cell types reflecting the disease mechanism [19].

Our 3D imaging approach also revealed depth-dependent orientation shifts in both fibroblasts and collagen fibers. These whorled, rotational structures were common across samples but appeared more coherent and organized in the control and HI OI groups than in the DN and EDS groups. The 3D orientation index and pixel-level correlation analysis confirmed that fibroblast orientation was tightly coupled to collagen fiber alignment (correlation coefficients ~0.9), underscoring the central role of fibroblasts in matrix organization. Notably, control cell lines, particularly neonatal, displayed more stable orientation patterns, while the DN OI and EDS groups exhibited broader angular distributions and more disordered fiber architecture. Individual collagen α-chains adopt a left-handed helical conformation, which assemble into a right-handed triple helix before incorporation into fibrils [25]. The presence of fibrils with differing twist directions, or a mixture of assembled fibrils and unincorporated chains, may indicate aberrant folding or defects in the assembly process seen in collagen-structure defects, common for DN OI and EDS.

Interestingly, the spatial distribution of collagen within the 3D culture varied between groups. In the EDS group, collagen was primarily deposited at intermediate depths, while control, HI OI, and DN OI cultures showed more continuous distributions. These differences in collagen localization and matrix anisotropy may reflect fundamental alterations in secretion dynamics, cell–matrix feedback, or matrix remodeling capabilities among the disease groups.

Taken together, our findings demonstrate that in 3D fibroblast models, both the quantity and quality of collagen, as well as fibroblast morphology and organization, are markedly altered in OI and EDS. These alterations may mirror the tissue-level manifestation of the diseases, underscoring the physiological relevance of our model framework. Beyond matrix abnormalities, such cellular and extracellular changes can likely contribute to the tissue-level fragility observed in these diseases. In particular, the larger, rounder morphology of HI fibroblasts and the elongated, narrow shapes of DN and EDS fibroblasts relative to controls indicate cell-intrinsic phenotypic differences that could influence ECM deposition, alignment, and mechanical integrity.

One of the study’s limitations is the variation in donor ages across different disease groups [26,27]. There was a clear effect of age on collagen production and organization. Neonatal control fibroblasts secreted more collagen and formed thinner, more uniformly aligned fibers than adult controls, indicating that aging may impair collagen biosynthesis and alter matrix structural properties. Fibroblast morphology and the ability to produce ECMs is dependent on age. Neonatal dermal fibroblasts displayed a stretched, spindle-like morphology, and produced abundant, well-organized, aligned collagen fibers. Adult fibroblasts adopt a more collapsed morphology and generated reduced collagen with disorganized fiber orientation and increased fragmentation. These age-dependent differences in fibroblast form, abundance, and ECM architecture could potentially mask differences in disease phenotype of both OI and EDS cell lines, emphasizing the importance of age-matching in in vitro studies of connective tissue disorders.

Secondly, this study focuses on unmineralized ECMs; however, in the context of OI and EDS, the mineralized ECM found in bones and teeth is of greater relevance, as it underlies one of the primary clinical disease manifestations. Mineral deposition within collagen fibrils modifies their mechanical properties, increasing rigidity while reducing flexibility, and alters both the optical and density characteristics of the tissue [28]. Therefore, evaluating cell–matrix interactions within a mineralized ECM model would be a critical direction for future investigations.

While our 3D model represents a significant advancement over conventional 2D culture systems, it remains a simplified representation of the native tissue environment. To further enhance its physiological relevance, in future studies we will incorporate additional ECM components such as fibronectin and elastin, the application of mechanical stimulation to simulate tissue loading, or co-culture systems with other relevant cell types, such as immune or endothelial cells. These additions could provide a more comprehensive view of the cell–matrix interactions that underlie tissue integrity and the dynamic environment underlying disease progression. Moreover, the use of longitudinal live-cell imaging with HHGM offers exciting potential for capturing dynamic processes such as matrix deposition, fiber remodeling, and fibroblast migration in real time [29].

Notably, HHGM can also be applied to label-free dynamic imaging of unprocessed live tissue [30], enabling real-time visualization of biopsied samples from OI and EDS patients. Establishing the physiological relevance of our methodology with the current study opens opportunities for the dynamic, non-destructive analysis of disease-relevant tissue architecture and cellular behavior within their intact native matrix. Such an approach could significantly advance our ability to study patient-specific disease mechanisms and therapeutic responses ex vivo, bringing us closer to functional diagnostics and personalized treatment strategies for connective tissue disorders.

Finally, linking in vitro phenotypes to clinical severity, such as bone fragility, vascular complications, or joint hypermobility, could identify potential cellular or ECM biomarkers for disease progression and response to therapy in OI and EDS.

## 4. Materials and Methods

### 4.1. Ethics Statement

Skin biopsies were acquired from the inner arm of donors after informed consent according to the medical ethics committee of the VU University.

### 4.2. Three-Dimensional Fibroblast Culture

Fibroblast cell lines from the four investigated groups were used for the 3D culture model. Fibroblast cultures of controls (from neonatal and adult donors), HI (from adult donors), DN (from neonatal donors), and EDS (from young donors) were established from dermal punch biopsies. The age, gender, and other information of donors at biopsy acquisition can be found in Table 1. Dermal fibroblasts were cultured in Ham’s F-10 Nutrient Mix medium (Gibco, Grand Island, NY, USA, 31550-031) supplemented with heat-inactivated 10% fetal bovine serum (FBS) (Gibco, Grand Island, NY, USA, 10270-106) and 1% penicillin/streptomycin (P/S) (Gibco, Grand Island, NY, USA, 15140-122) at 37 °C with 5% CO_2_ in a humidified atmosphere. Cell lines tested negative for mycoplasma.

Prior to the experiments, cells were switched to the Dulbecco’s Modified Eagle Medium (DMEM) (Gibco, Grand Island, NY, USA, 41966029) supplemented with heat-inactivated 10% FBS and 1% P/S. Cells were seeded with a density of 70,000 cells per chamber of 4-well Nunc™ Lab-Tek™ II Chambered Coverglasses (Thermo Fisher Scientific Inc., Waltham, MA, USA, 155360), as described previously [3]. From the second day after cell seeding, each well was refreshed with 0.8 mL medium supplemented with freshly prepared 100 µg/mL 2-Phospho-L-ascorbic acid trisodium salt (Sigma-Aldrich, St. Louis, MO, USA) 3 times per week for 5 weeks. During the last 1–2 weeks before the measurement, the ascorbic acid concentration was lowered to 50 µg/mL to prevent cells detaching from the dish bottom (Supplementary Table S1).

### 4.3. HHGM Imaging

The fibroblast and secreted collagen were visualized by higher harmonic generation microscopy (HHGM). A home-built HHGM with forward-detected second and third harmonic generation (SHG, THG) and epi-direction detected 2-photon excited autofluorescence (2PEF), as shown in Figure 1a, was used for imaging of the secreted collagen, fibroblasts, cell nuclei, and cytoplasm, respectively (Figure 1b). THG is an optical interface-sensitive technique which visualizes cells and cell nuclei. SHG is generated by collagen and 2PEF visualizes the cytoplasm through the major endogenous fluorophore intracellular nicotinamide adenine dinucleotide (NADH) [31,32]. The forward-generated SHG and THG configuration was chosen because in thin cell cultures this signal was higher than in the epi-direction, which is commonly used for thicker tissue samples [33,34]. The repetition rate of the femtosecond pulsed laser (FSP-03, Seed Lasers, Vilnius, Lithuania) (85 fs, 13.3 MHz) with center wavelength of 1050 nm was reduced to 1 MHz by a pulse picker acousto-optic modulator (AOM) to keep a low average power of 5 mW, while achieving the high peak power necessary to generate the nonlinear signals. The laser pulses were focused on the sample with the objective Nikon 40× Oil CFI Super Fluor (N.A. 1.30, W.D. 0.22 mm, Tokyo, Japan), resulting in an optical resolution of 0.4 × 0.4 × 2.4 µm^3^. Two-dimensional images were obtained by scanning the laser beam over the sample with a pair of galvanometer mirrors controlled by a LabVIEW program (Flash Pathology B.V., Amsterdam, The Netherlands), and 3D images were obtained by moving the motorized stage holding the culture incubator in z direction relative to the objective [33]. The generated 2PEF was filtered from the 1050 nm fundamental photons by a dichroic mirror (DM1, FF872-Di01, Semrock, Rochester, NY, USA). The generated THG and SHG signals were collected with a condenser (CD, ACL2520U-A, Thorlabs, Newton, NJ, USA), then divided into two channels by a dichroic mirror (DM2, DMLP425R, Thorlabs, Newton, NJ, USA), and filtered by two bandpass filters (F1, 525nm CWL/15nm Bandwidth, #86-354, Edmund optics, Barrington, IL, USA; F2, FF01-355/40-25, Semrock, Rochester, NY, USA). We used a narrow 15 nm bandwidth SHG filter to avoid a 2PEF signal from the cytoplasm leaking into the SHG channel. Three photomultiplier tubes (PMT1, H10721-20; PMT2, H16201-40; PMT3, H10721-210; Hamamatsu, Naka-ku, Japan) were used to detect the signals. In the forward direction, the condenser, dichroic mirror, two filters, and two PMTs were installed on a portable mechanical arm, which was aligned with a reference position and fixed during the measurement (Figure 1a). The 3D fibroblast culture was kept in a stage-top incubator (Okolab, Pozzuolli, Italy, H301-K-Frame) with insert LABTEK-II-35-M under 5% CO_2_ and 37 °C with suitable humidity during the HHGM measurement; the positions of the insert for 35 mm circle dishes were sealed by tinfoil and scotch tape. Before the HHGM measurement, the culture medium in each well of the culture chamber was changed to Earle’s Balanced Salt Solution (EBSS) (Gibco, Grand Island, NY, USA, 14155048) with 1% P/S to reduce the autofluorescence noise caused by the phenol red in the culture medium, as shown in the third well of the culture dish in Figure 1a.

### 4.4. Fibroblast Segmentation

Fibroblast cell count was obtained by a fibroblast segmentation algorithm. We implemented an integrated workflow to segment the fibroblasts revealed by THG imaging, as described previously [35]. In this workflow, a THG image of fibroblasts was first pre-processed by histogram truncation to remove pixel outliers with high THG signals to enhance the image’s contrast. After image enhancement, the pre-processed image was further denoised by a tensor regularized total variation model proposed for THG microscopy, to fully remove the image noise. The denoised THG image was then segmented with an active contour model weighted by prior extremes at the pixel level to detect the fibroblasts. In order to separate overlapping fibroblasts, we used a watershed approach based on distance transform and morphological filters. This workflow enabled accurate detection and counting of fibroblasts.

### 4.5. Collagen Amount and Collagen Fiber Diameter

The collagen amount and collagen fiber diameter were analyzed by software for comparison between the four groups. All z-stacks of 3D fibroblast cultures were measured with the same laser power lower than 5 mW and similar forward-direction SHG PMT gain settings for collagen. The collagen amount of each z-stack was determined as the count of pixels with SHG intensity higher than a threshold value and was counted by a customized Matlab Version R2024a (Mathworks Inc., Natick, MA, USA) code. The threshold was decided by averaging the mean intensity of multiple local background areas in the z-stacks of the different cell groups.

For measuring the diameter of collagen fibers, the SHG intensity in all z-stacks was first enhanced by contrast-limited adaptive histogram equalization (CLAHE) by a customized Matlab code. Three images with visible single collagen fibers were selected from each z-stack, while three single collagen fibers per image were randomly selected and their diameter was measured manually by ImageJ Version 1.54f (National Institutes of Health, Madison, WI, USA). First, the straight-line tool was drawn perpendicular to the single collagen fiber; then, the SHG intensity profile along this straight line was fitted with a Gaussian function and the FWHM value was taken as the collagen fiber diameter. Collagen fibers of varying diameters and different SHG intensities were included. When the same fiber appeared on two adjacent images of the z-stack, the image with wider fiber diameter and stronger SHG signal intensity was selected for the measurement.

### 4.6. Fibroblast Area and Shape

Fibroblast morphology was measured manually using ImageJ for comparison between the four investigated groups. For measuring the cell area and width-to-length ratio, each two adjacent THG images in the z-stacks of fibroblasts were maximally projected into one image along the z direction, then the fibroblasts’ intensity was enhanced by CLAHE by a customized code in ImageJ. The z-projected THG images of the fibroblasts were used for cell morphology measurements, except for the several cell layers near the bottom and top of the culture dish, where cell areas showed clear deviations. A quarter of each z-projected THG z-stack with clear and complete fibroblast cell boundaries were randomly selected in the x–y plane. The cell area and width-to-length ratio of 2 to 3 complete fibroblasts per image were measured manually using ImageJ. Thin filopodia were not traced to avoid overestimation of cell area [11]. For each z-stack, between 14 and 72 fibroblasts with defined and complete cell shape were randomly selected for measurement.

### 4.7. Orientation Calculation

For comparing the orientation difference between the four investigated groups, the 3D orientation of fibroblast and collagen was calculated. THG and SHG images from all z-stacks used for 3D orientation calculation of fibroblasts and collagen fibers were first enhanced by CLAHE by a customized Matlab code. Then, the 3D orientation was calculated for each pixel in all z-stacks using a Matlab code reported by Liu, Z. et al. [12]. In brief, the dark intensity thresholds of fibroblasts and collagen for the calculation were decided by averaging the mean signal intensity of several background areas in the z-stacks of different cell groups separately. Both the azimuthal angle ϑ and polar angle φ were calculated; with main focus on the azimuthal angle ϑ of the fibroblasts and collagen as the 3D cell cultures were thin and φ did not differ substantially between different cell groups. The definitions of ϑ and φ are shown in Supplementary Figure S2b. The 3D orientation index for each z-stack of fibroblasts and collagen was also calculated based on the ϑ and φ value of each pixel in the z-stacks. When the 3D orientation index was close to 1, it indicated that the fibers are highly aligned, while a value close to 0 was indicative of randomly disordered direction [33].

### 4.8. Statistical Analysis

Data from 4 different groups were shown as mean ± SD (standard deviation) of the mean (except in Supplementary Figure S2a where data are depicted as mean ± SE (standard error) of the mean), compared statistically using One-way ANOVA followed by Tukey’s test for normal distribution, and Kruskal–Wallis ANOVA followed by Dunn’s test for not-normal distribution. OriginPro 2025 software (OriginLab Corporation, Northampton, MA, USA) was used for statistical analysis. The normality test of data were calculated by OriginPro normality test tool with 6 tests of Shapiro–Wilk, Kolmogorov–Smirnov, Lilliefors, and Anderson–Darling, etc.; *p* value < 0.05 indicated a significant difference during the multiple comparison.

## 5. Conclusions

In this study, we demonstrate the potential of label-free HHGM, combining forward-detected THG and SHG, for high-resolution, volumetric imaging of live 3D skin fibroblast cultures and their collagen matrices derived from both healthy individuals and patients with OI and EDS.

We identified disease-consistent alterations in collagen quantity, fiber diameter, spatial organization, and fibroblast morphology. HI OI is characterized by altered fibroblast size and shape, reduced collagen quantity, and a depth-dependent counterclockwise rotational pattern of collagen fibers and fibroblasts. This pattern is less prominent in DN OI and EDS. Irregular fiber organization was also observed in HI OI cell cultures. DN OI similarly shows collagen reduction but presents elongated fibroblasts with a less consistent orientation shift across z-layers. EDS exhibits elongated fibroblasts, collagen fiber thinning, and altered spatial collagen deposition predominantly localized at intermediate depths. Fibroblast orientation is tightly coupled to collagen fiber alignment in all groups.

These findings reflect the diverse pathological mechanisms involving ECM and fibroblast abnormalities, highlighting the biological relevance of 3D in vitro models combined with advanced HHGM imaging while maintaining model viability during measurements. This high-resolution, non-destructive, label-free platform enables detailed investigation of ECM dysregulation and cellular alterations in connective tissue disorders while benefiting from easy operation and fast imaging of the tissue’s ultra-structure. As such, it has the potential to provide a powerful tool for future research on live imaging of collagen synthesis, fibroblast dynamics, and drug responses in patient-specific models, in steps towards advancing personalized medicine for these conditions.

## Figures and Tables

**Table 1:** Information of healthy controls (C) and patients (P) for the culture of dermal fibroblast cell lines. The gender, age at biopsy acquisition, clinical indication, and pathogenic variant of each healthy control cell line and patient cell line including osteogenesis imperfecta (OI) Sillence type 1, 3, 4, and Ehlers–Danlos Syndrome (EDS) subtype 8 are listed.

Group	Samples ID	Gender	Age	Diagnosis	Pathogenic Variant
Control	C1	Male	44	Healthy control	NA
Control	C2	Male	34	Healthy control	NA
Control	C3	Male	0	Healthy control	NA
Control	C4	Male	0	Healthy control	NA
HI OI	P1	Female	29	Osteogenesis Imperfecta type 1	NM_000088.4(COL1A1):c.495T>A, p.(Tyr165*)
HI OI	P2	Male	38	Osteogenesis Imperfecta type 1	NM_000088.4(COL1A1):c.2784del, p.(Gly929Alafs*179)
DN OI	P3	Female	0	Osteogenesis Imperfecta type 4	NM_000088.4(COL1A1):c.1678G>A, p.(Gly560Ser)
DN OI	P4	Female	0	Osteogenesis Imperfecta type 3	NM_000089.4(COL1A2):c.2113_2121del, p.(Ala705_Pro707del)
EDS	P5	Male	11	Ehlers–Danlos Syndrome subtype 8	NM_000302.4(PLOD1):c.1651-2A>G, p.? ^a^

^a^ p.? means that there were no studies on protein level to confirm the effect of the pathogenic variant. Most likely, this is splice site pathogenic variant resulting in a null allele (absence of protein product), with no experimental proof. According to the nomenclature it is stated as p.?.

## Data Availability

The original contributions presented in this study are included in the article/Supplementary Material. Further inquiries can be directed to the corresponding authors.

## Supplementary Materials

The following supporting information can be downloaded at: https://www.mdpi.com/article/10.3390/ijms262411848/s1.
