# Supplementary material for: Assessment of Collagen and Fibroblast Properties via Label-Free Higher Harmonic Generation Microscopy in Three-Dimensional Models of Osteogenesis Imperfecta and Ehlers-Danlos Syndrome
Source: Int J Mol Sci. 2025 Dec 8;26(24):11848. doi: 10.3390/ijms262411848 (PMC12732449; doi:10.3390/ijms262411848)
Supplement: Supplementary file 1 [file ijms-26-11848-s001.zip › ijms-3910195-supplementary.pdf]

Supplementary Material

Supplementary figure titles and legends

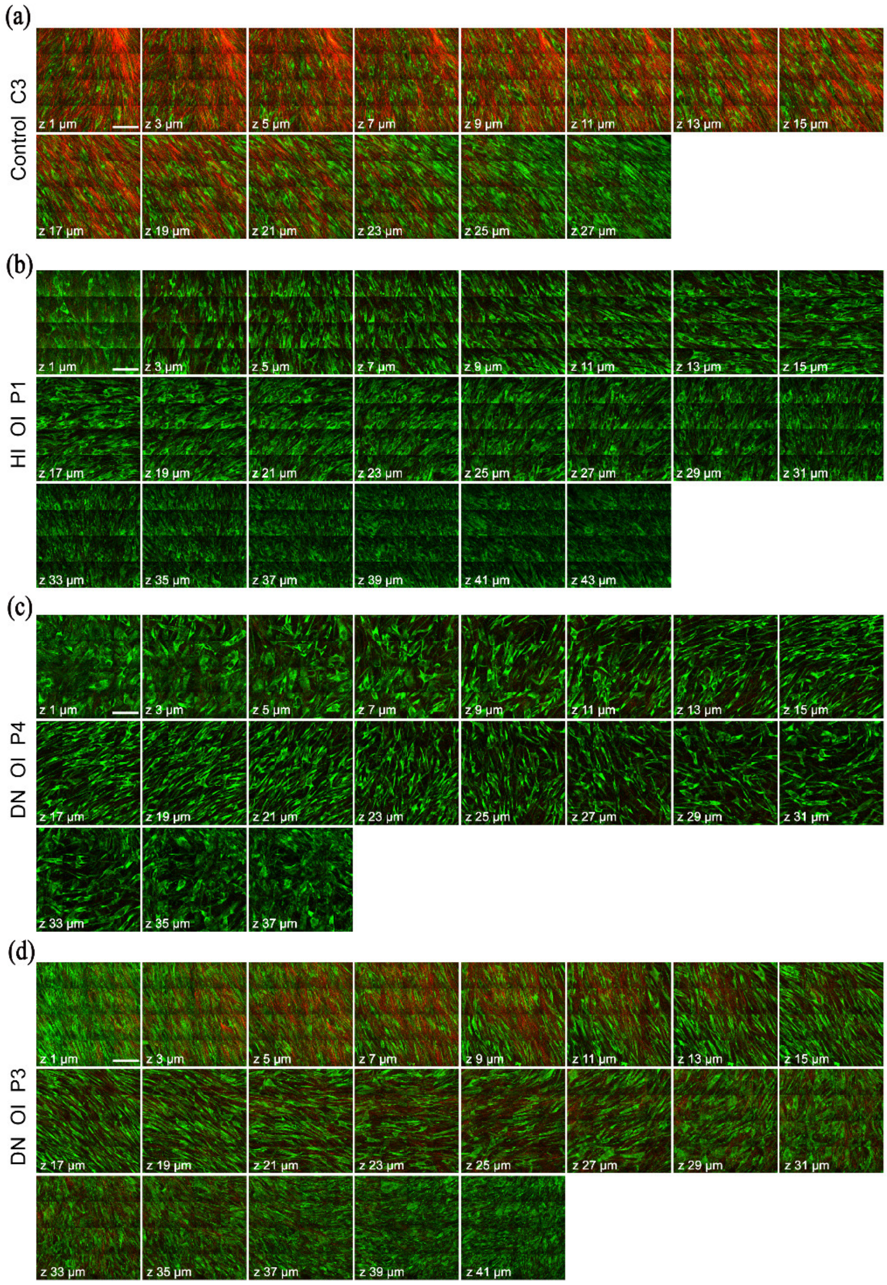

Supplementary Figure S1 | Representative local area of z-stacks of other neonatal control from donor C3, HI from donor P1, DN from donor P3 and P4 cell lines. All images for quantification were measured in high quality mode (5 pixels/μm). a, Zoom-in local area of the z-stack of one neonatal control cell line from donor C3. Scale bar, 100 μm. b, Zoom-in local area of the z-stack of one HI cell line from donor P1. Scale bar, 100 μm. c, Zoom-in local area of the z-stack of one DN cell line from donor P4. Scale bar, 100 μm. d, Zoom-in local area of the z-stack of another DN cell line from donor P3. Scale bar, 100 μm.

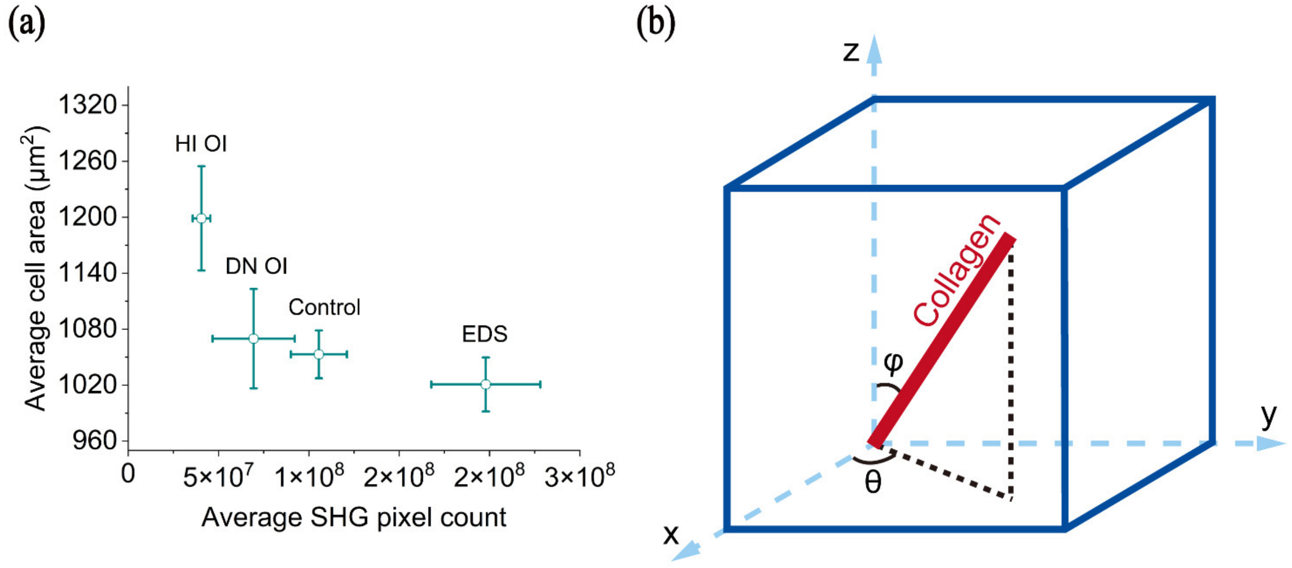

Supplementary Figure S2 | The angle definition and correlation of cell area and collagen amount. a, The average cell area and average total SHG pixel count of all z-stacks in four cell groups. Data shown as mean ± SE. b, The definition of azimuthal angle ϑ and polar angle φ in 3D space for the orientation calculation of collagen and fibroblasts in 3D culture.

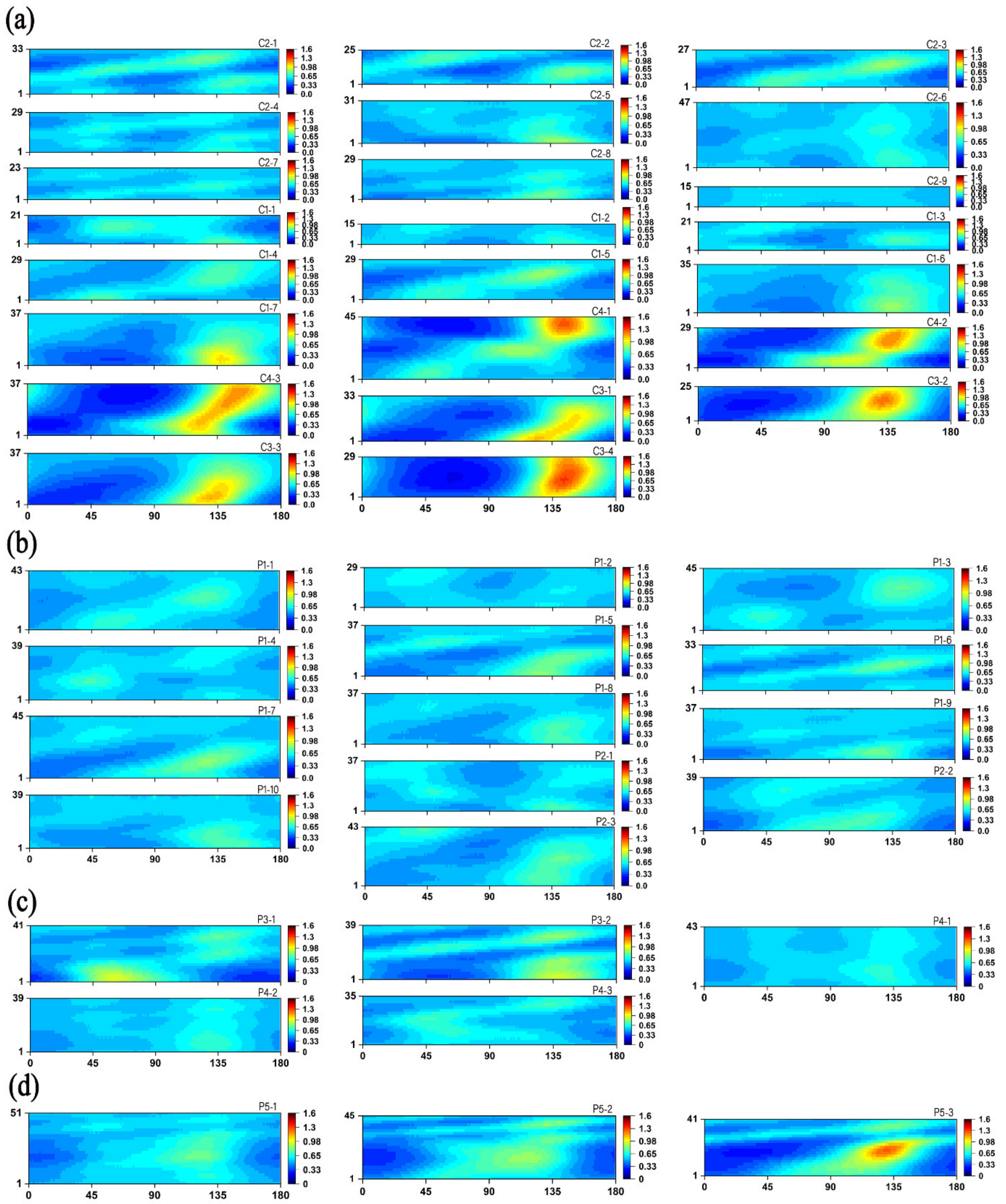

Supplementary Figure S3 | ϑ directional orientation heatmaps of collagen for all z-stacks of four cell groups, shown by percentage of pixel count. Along x axis is ϑ angle distribution in the range between 0° and 180°, across y axis is the ϑ information at each z layer (the z direction distance from culture dish bottom) with 2 μm z interval. The redder the color, the more pixels are at this angle, as indicated by the right scale bar. a, Control group with 4 cell lines from adult donors C1 and C2, and from neonatal donors C3 and C4. b, HI group with 2 cell lines from adult donors P1 and P2. c, DN group with 2 cell lines from neonatal donors P3 and P4. d, EDS group with cell line from young donor P5.

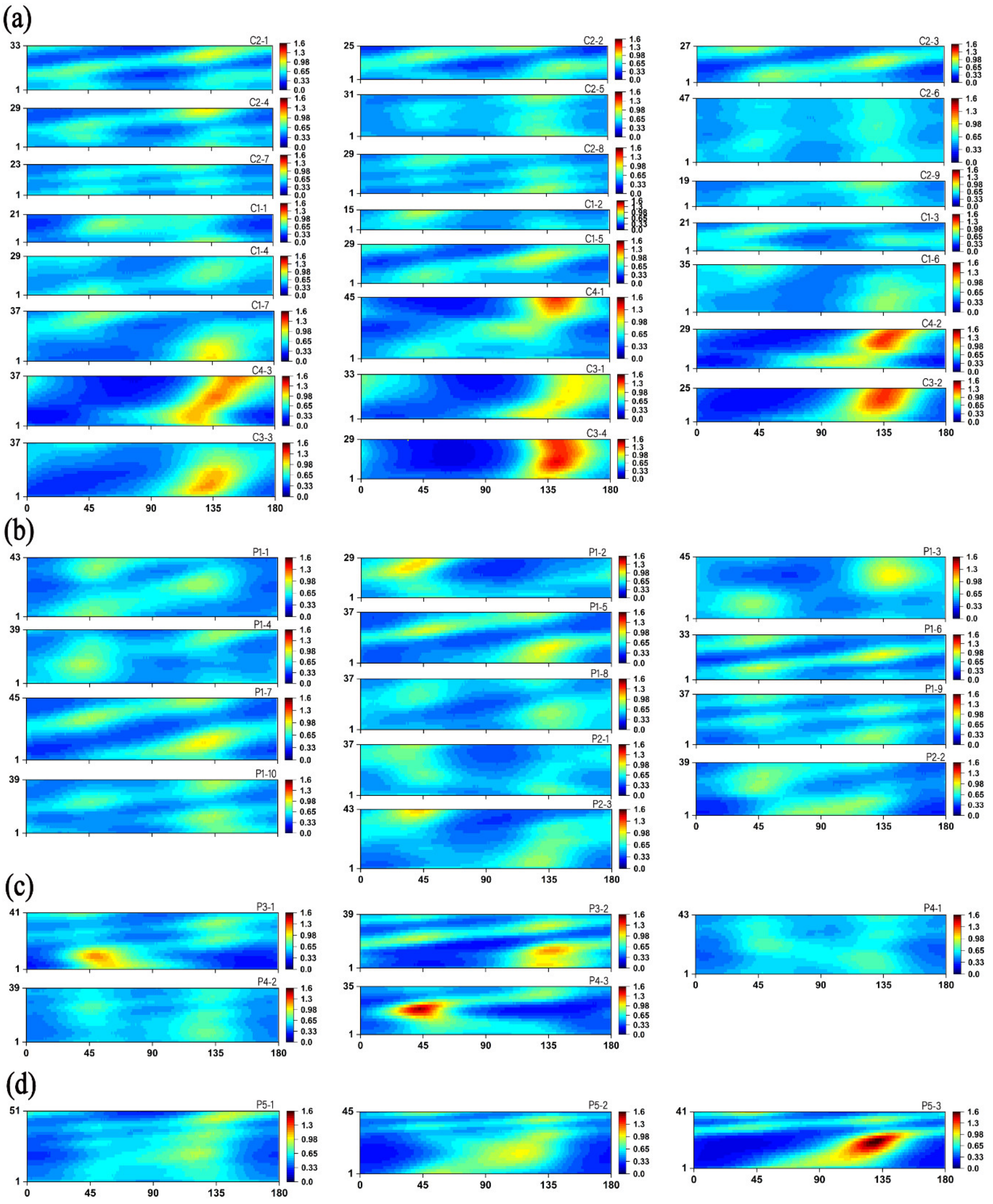

Supplementary Figure S4 | ϑ directional orientation heatmaps of fibroblasts for all z-stacks of four cell groups, shown by percentage of pixel count. Along x axis is ϑ angle distribution in the range between 0° and 180°, across y axis is the ϑ information at each z layer (the z direction distance from culture dish bottom) with 2 μm z interval. The redder the color, the more pixels are at this angle, as indicated by the right scale bar. a, Control group with 4 cell lines from adult donors C1 and C2, and from neonatal donors C3 and C4. b, HI group with 2 cell lines from adult donors P1 and P2. c, DN group with 2 cell lines from neonatal donors P3 and P4. d, EDS group with cell line from young donor P5.

Supplementary Table with title and legend

Supplementary Table S1: Location information of all cell lines in each 4-well Nunc™ Lab-Tek™ II Chambered Coverglasses. The cell lines in the same chamber means were seeded in different wells of the same chamber on the same day, refreshed culture medium with same concentration of 2-Phospho-L-ascorbic acid trisodium salt, same cultivation time, measured on the same day by HHGM with same settings. In each chamber, two same cell line numbers mean two positions in the same well were measured 1×1 mm^2 FOV z-stacks with 2 μm z interval.

**Chamber**	**Cell lines**						
**Chamber 1**	Control C1-3	Control C1-4	Control C2-5	Control C2-6	HI OI P1-5	HI OI P1-6	
**Chamber 2**	Control C1-5	Control C1-6	Control C2-7	Control C2-8	HI OI P1-7	HI OI P1-8	
**Chamber 3**	Control C1-7	Control C2-9	HI OI P1-9	HI OI P1-10			
**Chamber 4**	EDS P5-2	EDS P5-3	Control C2-1	Control C2-2	HI OI P1-1	HI OI P1-2	
**Chamber 5**	EDS P5-1	Control C2-3	Control C2-4	HI OI P1-3	HI OI P1-4		
**Chamber 6**	DN OI P3-1	DN OI P3-2	Control C3-1	Control C3-2			
**Chamber 7**	Control C3-3	Control C3-4					
**Chamber 8**	Control C4-1	Control C4-2	Control C1-1	HI OI P2-1	HI OI P2-2	DN OI P4-1	DN OI P4-2
**Chamber 9**	Control C4-3	Control C1-2	HI OI P2-3	DN OI P4-3			
